# Supplementary material for: Evaluation of ultrasound as diagnostic tool in patients with clinical features suggestive of carpal tunnel syndrome in comparison to nerve conduction studies: Study protocol for a diagnostic testing study
Source: PLoS One. 2023 Nov 10;18(11):e0281221. doi: 10.1371/journal.pone.0281221 (PMC10637656; doi:10.1371/journal.pone.0281221)
Supplement: S2 Checklist — (DOCX) [file pone.0281221.s002.docx]

**STARD for Abstracts: essential items for reporting diagnostic accuracy studies in journal or conference abstracts**

| Section | Item | Text |
| --- | --- | --- |
|  | Identification as a study of diagnostic accuracy using at least one measure of accuracy (such as sensitivity, specificity, predictive values, or AUC) | To calculate the sample size (EPIDAT program) we proposed a sensitivity of 78% and specificity of 87% with a confidence level of 95%, requiring 438 patients (264 NPT positive, 174 NPT negative). |
| **Background and objectives** | Study objectives | The main objective of this diagnostic test evaluation study is to investigate the value of ultrasound in the diagnosis of CTS, and among the secondary objectives, to establish the ultrasound parameters that are predictors of CTS in comparison with neurophysiological studies, attempting to standardize a protocol and reference values that determine the presence or absence of CTS. |
| **Methods** | Data collection: whether this was a prospective or retrospective study | Prospective, cross-sectional study. |
|  | Eligibility criteria for participants and settings where the data were collected | Patients will come consecutively from the Neurophysiology Department of the Virgen Macarena Hospital, with clinical suspicion of CTS and fulfilling the inclusion/exclusion criteria. |
|  | Whether participants formed a consecutive, random, or convenience series | Patients will come consecutively from the Neurophysiology Department of the Virgen Macarena Hospital, with clinical suspicion of CTS and fulfilling the inclusion/exclusion criteria. |
|  | Description of the index test and reference standard | The reference test with which we compared the ultrasound is the neurophysiological test (NPT). We followed an ultrasound study protocol that included the ultrasound variables: cross-sectional area at the entrance and exit of the tunnel, range of nerve thinning, wrist-forearm index, flexor retinaculum bulging, power Doppler uptake and the existence of adjacent wrists or masses. |
| **Results** | Number of participants with and without the target condition included in the analysis | 264 NPT positive, 174 NPT negative |
|  | Estimates of diagnostic accuracy and their precision (such as 95% confidence intervals) | To calculate the sample size (EPIDAT program) proposed a sensitivity of 78% and specificity of 87% with a confidence level of 95% |
| **Discussion** | General interpretation of the results | The use of ultrasound as a diagnostic tool in CTS has many advantages for both doctors and the patients, as it is a non-invasive, convenient, and fast tool increasingly accessible to professionals. |
|  | Implications for practice, including the intended use of the index test | The use of ultrasound as a diagnostic tool in CTS has many advantages for both doctors and the patients, as it is a non-invasive, convenient, and fast tool increasingly accessible to professionals. |
| **Registration** | Registration number and name of registry | Evaluation of ultrasound as diagnostic tool in patients with clinical features suggestive of carpal tunnel syndrome in comparison to nerve conduction studies: study protocol for a diagnostic testing study: Retrospectively registered |

*Cite this as: Cohen JF, Korevaar DA, Gatsonis CA, Glasziou PP, Hooft L, Moher D, Reitsma JB, de Vet HCW, Bossuyt PM, for the STARD Group. STARD for Abstracts: Essential items for reporting diagnostic accuracy studies in journal or conference abstracts. BMJ 2017;358:j3751*
