# Supplementary material for: Evaluation of ultrasound as diagnostic tool in patients with clinical features suggestive of carpal tunnel syndrome in comparison to nerve conduction studies: Study protocol for a diagnostic testing study
Source: PLoS One. 2023 Nov 10;18(11):e0281221. doi: 10.1371/journal.pone.0281221 (PMC10637656; doi:10.1371/journal.pone.0281221)
Supplement: S1 File — (PDF) [file pone.0281221.s003.pdf]

SOLE OPINION IN THE AUTONOMOUS COMMUNITY OF ANDALUSIA

D/Dª: Carlos García Pérez as secretary of the CEI of the Vírgen Macarena-Virgen del Rocío university hospitals

CERTIFIES

That this Committee has evaluated the proposal of the promoter / researcher (There is no associated promoter) to carry out the study research titled:

TITLLE OF THE STUDY:	EVALUATION OF ULTRASOUND AS A DIAGNOSTIC TOOL IN PATIENTS WITH CLINICAL FEATURES SUGGESTIVE OF CARPAL TUNNEL SYNDROME IN COMPARISON TO NERVE CONDUCTION STUDIES: STUDY PROTOCOL FOR A DIAGNOSTIC TESTING STUDY.
Protocol, version:	1
HIP, version:	1
CI, version:	1

And that considers that:

The necessary requirements for the suitability of the protocol in relation to the objectives of the study are met and it conforms to the principles ethics applicable to this type of study.

The capacity of the investigator and the means available are appropriate to carry out the study.

The foreseeable risks and inconveniences for the participants are justified.

That the economic aspects involved in the project do not interfere with the ethical principles.

And that this Committee considers, that said study can be carried out in the Centers of the Autonomous Community of Andalusia

that are related, for which it corresponds to the Directorate of the corresponding Center to determine if the capacity and means available are appropriate for conducting the study.

What I signed in Seville on 03/08/2020

D/Dª: Carlos García Pérez as secretary of the CEI of the Vírgen Macarena-Virgen del Rocío university hospitals.

Código Seguro De Verificación:	a462739eb0fd339704749b3269feed7855a3aecd	Fecha	08/03/2020
Normativa	Este documento incorpora firma electrónica reconocida de acuerdo a la Ley 59/2003, de 19 de diciembre, de firma electrónica.		
Firmado Por	Carlos García Pérez		
Url De Verificación	https://www.juntadeandalucia.es/salud/portaldeetica/xhtml/ayuda/verificarFirmaDocumento.iface/code/a462739eb0fd339704749b3269feed7855a3aecd	Página	1/2
